# The FIND Program: Improving Follow-up of Incidental Imaging Findings

**DOI:** 10.1007/s10278-023-00780-6

**Published:** 2023-02-09

**Authors:** Kaitlin M. Zaki-Metias, Jeffrey J. MacLean, Alexander M. Satei, Serguei Medvedev, Huijuan Wang, Christopher C. Zarour, Paul J. Arpasi

**Affiliations:** 1 Department of Radiology, Trinity Health Oakland Hospital/Wayne State University School of Medicine, Pontiac, MI USA; 2 Huron Valley Radiology, Ypsilanti, MI USA

**Keywords:** Incidental findings, Incidental imaging findings, Computed tomography, Artificial intelligence, Natural language processing, Natural language understanding

## Abstract

Incidental findings are findings identified on imaging which are unrelated to the original reason for examination and require follow-up. The Radiology Finding Incidental Disease (FIND) Program was designed to track and improve follow-up of incidental imaging findings. The purpose of this study was to determine the frequency of incidental findings on cross-sectional imaging and the adherence to suggested follow-up of incidental findings prior to and after implementation of a structured reporting and tracking system. A retrospective analysis of 2000 patients with computed tomographic cross-sectional imaging was performed: 1000 patients prior to implementation of the FIND Program and 1000 patients 1 year after establishment of the program. Data collected included the frequency of incidental findings, inclusion of follow-up recommendations in the radiology report, and adherence to suggested follow-up. There was a higher rate of completion of recommended follow-up imaging in the post-implementation group (34/67, 50.7%) compared to the pre-implementation (16/52, 30.8%) (*p* = 0.03). Implementation of an incidental findings tracking program resulted in improved follow-up of incidental imaging findings. This has the potential to reduce the burden of clinically significant incidental findings possibly resulting in later presentation of advanced disease.

## Introduction

### Incidental Findings and Communication

In recent years, imaging has become widely used across all medical specialties for the diagnosis and treatment of various conditions. With an ever-increasing volume of imaging studies being ordered and wider accessibility of more advanced imaging, there is a proportionately expected increase in incidental imaging findings [1]. Incidental findings are findings that do not pertain to the original queried reason for the exam but require timely follow-up; they encompass many findings including nodules, masses, indeterminate lesions, and vascular aneurysms. Incidental findings may be found in approximately up to one-third of imaging studies, depending on the modality [1]. Conventionally, ordering physicians are responsible for communicating findings to the patient and arranging appropriate follow-up where necessary, either by ordering the recommended follow-up investigation or communicating with the physician who will be caring for the patient going forward; however, radiologists and radiology departments may feel a degree of shared responsibility for ensuring this information is conveyed to the patient. Incidental findings may be detected on imaging obtained during an emergency room visit or hospitalization, where the ordering physician is not the patient’s primary care physician and will likely not be responsible for ensuring follow-up of the finding. Issues may arise when the ordering physician is not the primary care physician, or when the patient does not have a designated primary care physician.

A 2019 study by Hammer et al. evaluated the effectiveness of closed-loop communication between radiologists and ordering physicians regarding follow-up of incidental pulmonary nodules (IPNs) via implementation of a tracking system [2]. This allowed the ordering physician to schedule follow-up appointments and imaging studies based on the radiologist’s suggestions [2]. Lack of appropriate follow-up resulted in an alert being sent to the ordering provider. The results included an increase in clarity of follow-up recommendations, improved agreement to the proposed collaborative follow-up plan by radiologists, and an increase in engagement of the ordering physician in the follow-up process of IPNs [2].

### The Radiology Finding Incidental Disease (FIND) Program: An Overview

The Finding Incidental Disease (FIND) Program began on July 1, 2019, when it was piloted at Trinity Health Ann Arbor Hospital in the Trinity Health Michigan system (Appendix). It was an expansion of the previously established LungCare Program, which was implemented in combination with the lung cancer screening program in 2014 after a root cause analysis identified 14 patients with advanced stage lung cancer in 1 year due to nonadherence to recommended follow-up of incidental pulmonary nodules. As a result of these efforts, the majority of patients at our regional healthcare system are now being diagnosed with lung cancer at stages I and II, rather than stages III or IV. The FIND Program was expanded in September 2020, when it was implemented at Trinity Health Oakland Hospital.

### The FIND Program: Tracking Software

**Fig. 1:**
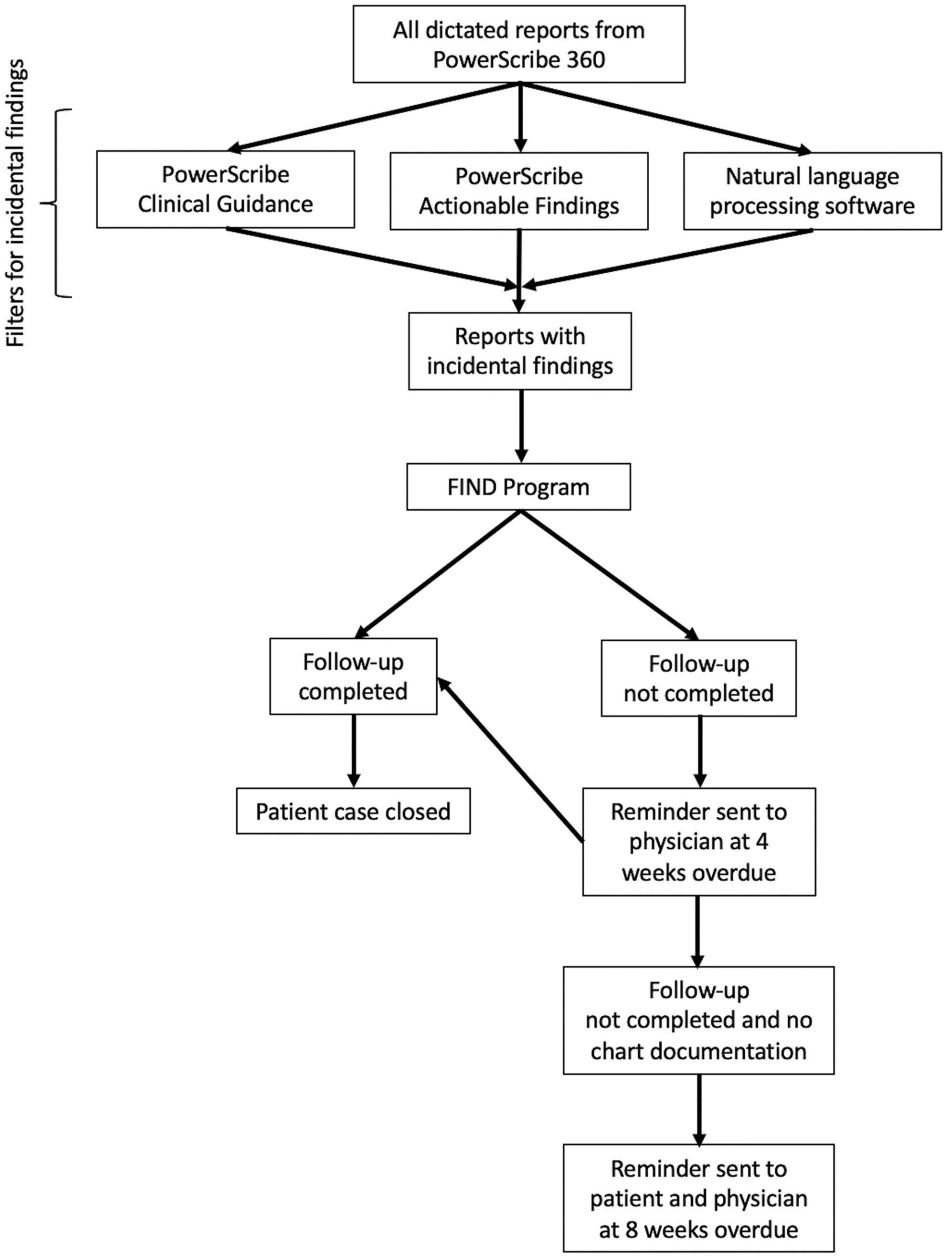
Schematic diagrammatic overview of the Radiology Finding Incidental Disease (FIND) Program

FIND is an electronic tracking software that is fed information from radiology reports. Incidental and unexpected findings are flagged for enrollment in the FIND Program and fed into the tracking software from the impression section of the radiology report and tools embedded within the voice-recognition dictation software (Nuance PowerScribe 360 | Reporting, London, England) via the Power Connect Actionable Findings (PCAF) and Clinical Guidance functions (Fig. 1).

The PCAF functionality allows radiologists to communicate critical or incidental findings to ordering providers directly through PowerScribe by sharing either the text from the report impression, customized text, or a voice note.

The Clinical Guidance function in PowerScribe is a decision support tool which allows radiologists to formulate structured recommendations by inputting the size, location, and characteristics or descriptors of an incidental finding. Follow-up recommendations for incidental findings are generated based on American College of Radiology white papers and can be auto-populated into the radiology report via the Clinical Guidance function in PowerScribe. Additionally, all radiologists are provided with PowerScribe Auto-Text macros for incidental findings, which can also be tracked by the FIND Program. Overall, the tools provided by PowerScribe are designed to streamline and standardize reporting of incidental findings and follow-up recommendations, and their use is ultimately dependent upon the radiologists. Every report in which PCAF or Clinical Guidance was used is automatically flagged for review by the FIND Program.

All radiology reports, except for mammography, are analyzed via natural language understanding (NLU) software to search for keywords and suspicious phrases in radiologist reports, such as “incidental”, “unexpected”, “could further evaluate”, “further assess”, “recommend/recommended”, and “advise/advised”. Furthermore, examples of nine different ways to state follow-up recommendations were introduced to the NLU to enhance capture of the incidental finding. Three primary parameters are noted by the NLU: the incidental finding, the recommended follow-up examination, and the timeframe in which follow-up is recommended to be completed.

### The FIND Program: Communication

A key component of the FIND Program is the emphasis on closed-loop communication. Nurse navigators embedded into the FIND Program monitor the tracking board of a patient’s unexpected or incidental findings and follow up with the primary care or ordering physician when the timeframe for recommended imaging follow-up is exceeded, and eventually follow up with the patient if needed. If the incidental finding is not included in the patient’s emergency department or inpatient discharge summary, the radiology report is still flagged by the FIND Program and is intended to prevent these patients from falling through the cracks; the patient’s primary care or ordering physician is notified or reminded of these incidental findings and the recommended follow-up at 4 weeks overdue. At 8 weeks overdue, a follow-up letter is sent to both the patient by US mail; patients are advised to call their primary care physician, given information to find a primary care physician if they do not have one, and given the contact information for the FIND Program nurse navigator for further information regarding their incidental findings. The ordering or primary care physician is also reminded of the incidental finding at 8 weeks overdue.

### Study Objectives

This study aims to investigate the effects of standardization of the reporting and tracking of incidental and critical imaging results with implementation of the Finding Incidental Disease (FIND) Program.

## Materials and Methods

A retrospective analysis of the volume of incidental findings, including the frequency of both identification and appropriate follow-up recommendations, and subsequent follow-up prior to and after implementation of the FIND Program at Trinity Health Oakland Hospital was performed. 

Electronic medical records and final reports of a total of 2000 randomly selected patients were investigated. One thousand patients with 1349 studies from January 2019 to January 2020 prior to implementation of the FIND Program and 1000 patients with 1267 studies from September 2020 to September 2021 1 year after implementation of the FIND Program were analyzed. Body imaging studies were designated as any CT of the chest, abdomen, or pelvis, including CT angiography, coronary calcium score CT, and low-dose chest CT for lung cancer screening. Imaging studies placed or performed as one order, such as CT of the chest, abdomen, and pelvis, were counted as one imaging study. Studies from all patient settings—emergency room, inpatient, and outpatient—were included. All reports were approved and signed by board-certified body imaging radiologists. Information was obtained from these final interpretation reports and not via review of the actual images.

Specific parameters that were analyzed prior to and after implementation of the FIND Program include the rate of identification of incidental imaging findings, follow-up recommendations, and completion of imaging follow-up. Statistical analysis was performed using the IBM SPSS statistics software version 25.0 (IBM, Armonk, NY). Fisher exact tests were used to compare outcomes from the pre-implementation and post-implementation groups with a *p*-value of < 0.05 for significance.

## Results

A total of 1349 studies were performed on 1000 patients from January 2019 to January 2020 prior to implementation of the FIND Program at Trinity Health Oakland Hospital, and 1267 studies were performed on 1000 patients from September 2020 to September 2021 1 year after implementation of the FIND Program. The mean age of patients was 60.9 ± 17.9 years, with 865 (43.1%) males and 1135 (56.9%) females; age and gender distribution were not significantly different between the pre-intervention and post-intervention groups (*p* = 0.46 and *p* = 0.53, respectively).

There was no significant difference in the rate of incidental findings reporting between the pre-implementation and post-implementation groups (Table 1). There was a statistically significant higher rate of follow-up study recommendation for incidental findings in the post-intervention group (67/70, 95.7%) compared to the pre-intervention group (52/69, 75.4%) (*p* = 0.001) (Table 1). Incidental findings noted on studies for emergency department (ED) patients more frequently recommended follow-up imaging in the post-intervention group (97.7%, 42/43) compared to 81.8% (27/33) of studies in the pre-intervention group (*p* = 0.04) (Table 2).

**Table 1:** Frequency of incidental finding detection, follow-up recommendation, and completed follow-up prior to and after implementation of the FIND Program

	***n*/*N***	**%**	***p*-value**
**Frequency of detection of incidental imaging findings**			
Pre-implementation	82/1274	6.4%	0.452
Post-implementation	70/1228	5.7%	
**Frequency of follow-up recommendations**			
Pre-implementation	52/69	75.4%	0.001
Post-implementation	67/70	95.7%	
**Completion of recommended follow-up imaging**			
Pre-implementation	16/54	29.6%	0.03
Post-implementation	34/67	50.7%

**Table 2:** Frequency of incidental finding detection, follow-up recommendation, and completed follow-up prior to and after implementation of the FIND Program across emergency department, inpatient, and outpatient settings

	Pre-implementation		Post-implementation		
	*n*/*N*	%	*n*/*N*	%	*p*-value
**Frequency of detection of incidental imaging findings**					
Emergency Department	38/543	7.0%	44/621	7.1%	1.000
Inpatient	20/259	7.7%	13/270	4.8%	0.208
Outpatient	24/472	5.1%	13/337	3.9%	0.496
**Frequency of follow-up recommendations**					
Emergency Department	27/33	81.8%	42/43	97.7%	0.038
Inpatient	12/18	66.7%	13/14	92.9%	0.104
Outpatient	13/18	72.2%	12/13	92.3%	0.359
**Completion of recommended follow-up imaging**					
Emergency Department	5/21	19.2%	22/40	55.0%	0.005
Inpatient	2/13	15.4%	6/14	42.9%	0.209
Outpatient	9/15	60.0%	6/13	46.2%	0.705

Patients in the cohort after implementation of the FIND Program had a higher rate of completing follow-up of 50.7% (34/67) in contrast with the pre-intervention group (16/52, 30.8%) (*p* = 0.03) (Table 1). Emergency department patients with incidental findings for which imaging follow-up was recommended had a higher rate of adherence to follow-up recommendations in the post-implementation group (22/40, 55.0%) compared to the pre-implementation group (5/26, 19.2%) (*p* = 0.01) (Table 2).

A total of two patients each in the pre-implementation group (2/1349; 0.1%) and post-implementation group (2/2167; 0.2%) were documented in the EMR to have been deceased prior to the end of the timeframe for recommended follow-up.

There was no significant difference in the outcomes of incidental findings follow-up between the pre-implementation and post-implementation groups. In the pre-implementation group, 7/21 (33.3%) of incidental findings were determined to be malignant, concerning for malignancy, or otherwise clinically significant, compared to 35.3% (12/34) in the post-implementation group (*p* = 1.0).

## Discussion

The results of this study indicate that the implementation of a structured tracking program for reported incidental imaging findings resulted in improved follow-up and adherence to recommended time intervals for imaging examinations. This study found statistically significantly improved rates of follow-up after implementation of the FIND program, including follow-ups within the recommended timeframe.

The benefits of identifying incidental findings are rooted in the idea that earlier diagnosis of a medical condition can result in improved outcomes. These benefits need to be considered against the number of cases which receive follow-up for benign findings, findings which do not alter clinical outcomes or are untreatable, and cases where treating the incidental finding results in adverse health outcomes [3]. Increased follow-up of incidental findings is most beneficial in organ systems which have the highest rate of malignancy, such as the breast [1]. Using evidence-based guidelines and evaluating the individual patient case when determining which incidental findings to follow-up will maximize the benefits of programs similar to FIND, while minimizing the consequences of unnecessary imaging. It is important to note that although the ACR white papers utilized in this study include recommendations for incidental findings across most major organ systems, these consensus guidelines are often flexible and used as suggestions for future follow-up, rather than providing evidence-based rigid timeframes [1].

In our study, the rate of benignity and malignancy of incidental findings which were followed up was relatively unchanged between the pre-intervention and post-intervention groups, although more patients underwent follow-up examinations in the post-intervention group. This suggests that, while overall follow-up rates improved after implementation of the FIND Program, there was no direct translation to increased detection of malignant or potentially malignant incidental findings, as the proportion remained similar between both groups.

Important in the process of communicating incidental findings is the way a radiologist communicates the report. The language used should clearly identify the incidental finding, recommend a modality of follow-up imaging, need for contrast, an appropriate time interval, and be placed in the impression section of the report [4, 5]. This has been shown to result in higher completion rates when compared to language which is less concise, indirect, or without clear recommendations [4]. The use of templated reporting compared to free-dictation can help maintain a clear structure and promote evidence-based and guideline-supported practices [6]. This is one method by which radiologists can ensure a higher likelihood of effectively communicating findings, and in turn ensuring higher chance of follow-up.

Interestingly, the success of the FIND Program in ensuring appropriate follow-up of incidental imaging findings was best observed in the emergency room setting. This can likely be attributed to the potential gap in care between the emergency room physician, who will initially hear of an incidental finding, and a patient’s primary care or specialist physician, who will ultimately assume care and be responsible for following up on an incidental finding. The results of this study indicate that programs such as FIND may have a more profound impact on imaging follow-up in emergency department settings, acting as a bridge between emergency physicians and outpatient primary care and/or specialist physicians in a patient’s continuity of care.

One barrier in the follow-up of incidental findings is concern for cost-effectiveness; as an increasing number of imaging studies are being performed, an increased cost is expected. In determining the cost-effectiveness of follow-up on incidental findings, the cost of follow-up imaging must be weighed against quality-adjusted life years (QALYs) [7, 8]. In addition to the financial considerations, follow-up imaging can further burden the healthcare system through other means. The psychological effect on patients who are made aware of incidental results can be significant. Patients have demonstrated a preference for face-to-face communication of incidental findings, which could carry further financial and scheduling burdens for doctors [9]. However, this form of communication is likely ideal, as it would improve patient understanding, and in turn reduce distress by allowing shared decision-making in the way results are communicated, clarification regarding the need for follow-up imaging, and allow the ordering physician to answer any patient questions or concerns.

Limitations of this study include the available methods of assessment for completion of follow-up, which was restricted by the information available in the integrated EMR. Reasons for loss of follow-up include an inability to contact the patient or their physician, the patient and/or physician choosing not to follow-up on the incidental finding, the patient may prefer to receive their care at a different hospital system, and patient relocation. Other reasons documented by the FIND Program when a patient does not receive follow-up imaging include “sufficient documentation in the electronic medical record”, patient or physician opted for surgical management of the incidental finding, or that the incidental finding is stable or unchanged based on additional reports or documentation available to the ordering or primary care physician. Follow-up may have been completed at an outside institution without shared information in the EMR, which the authors do not have access to records for. Furthermore, the authors were unable to reliably assess if a reason as to why follow-up was not completed was documented, as not all ordering physicians used the institution’s EMR. This does not account for patients that may have had an informed discussion with their physician and ultimately decided not to pursue follow-up imaging. The impact of socioeconomic factors and barriers to care may also play a role in loss of patient follow-up but is difficult to assess or quantify.

An additional limitation involves the frequency of incidental findings. Only 152 of the 2000 (7.6%) included patients in both the pre- and post-intervention groups were found to have incidental findings. In contrast, the frequency of incidental findings on CT images has previously been suggested by the literature to be around 31.1% [10]. One potential contributing factor is the subjectivity of the definition of an incidental finding and threshold for reporting said findings. Although inter-radiologist differences exist, the threshold for flagging an incidental finding for the FIND Program is determined by its potential clinical significance. Some studies included in the 2010 systematic review by Lumbreras et al. included minor and benign incidental findings in their analysis which would not typically warrant further evaluation, likely accounting for the difference in the frequency of reported incidental findings [10]. Furthermore, our study specifically evaluated incidental findings detected on thoracic, abdominal, and pelvic CT, and excluded neuroimaging, musculoskeletal imaging, and other modalities such as radiography, sonography, nuclear medicine, and magnetic resonance imaging. Additionally, our analysis excluded pulmonary nodules given the presence of our LungCare Program, which would have accounted for a total of 267/2000 (13.4%) incidental findings. Altogether, these factors likely account for the discrepancy in the rate of incidental findings in our study in comparison to the reported literature.

Further studies in the role of artificial intelligence (AI) in identifying and tracking incidental findings will provide insight into how radiologists can harness this technology to their advantage. The implications when applied to incidental findings are far reaching, including the potential for more efficient computer-aided detection (CAD). Natural language processing (NLP) and NLU systems are another form of AI which have been developed to identify key words in radiology reports which suggest the need for follow-up imaging. One study which used an NLP system to analyze 570,000 imaging studies found 29,000 lung-related follow-up recommendations; this information was integrated within the patient’s medical record, and provided alerts to both the patient and their care teams to ensure scheduling of follow-up imaging [11]. NLP systems have grown significantly over the past several years. Limitations related to ethics of sharing data and information availability, such as in cases when the radiology report does not include data and keywords the system searches for, can limit current applicability [12]. Furthermore, NLP systems do not consider a patient’s preference or automatically schedule follow-up imaging, and the responsibility to do so ultimately still lies with the ordering physician and the patient’s care team.

## Conclusion

Programs such as FIND can help improve follow-up rates for incidental imaging findings, including in the emergency room setting, by providing reminders to physicians and patients. This can lead to earlier detection of disease and should be communicated to the patient clearly and with regard for how this may affect a patient’s overall well-being. Determination of follow-up of incidental findings ultimately depends on the type and location of finding, the evidence-based guidelines regarding follow-up recommendations, and individual patient preferences.

## Appendix

The FIND Program is housed in the radiology department.

Ten years ago, a manual process was initiated as a result of a root cause analysis (RCA) for a patient returning with an advanced cancer. The RCA noted that the radiology report clearly stated the incidental finding (IF) and stated the concern for a malignancy and recommended a follow-up CT in 4–6 weeks.

The radiology report is the root of the IF and recommendations. The success and outcomes of the FIND Program are rooted in the radiologists providing the IF in the report. As such, the radiology department is best suited to monitor the outcomes, provide trending and feedback to the radiologists.

Nurse navigator staff are housed and budgeted to the radiology department. The FIND Program generates a high volume of follow-up imaging exams, with the highest volume being in CT, MRI, and ultrasound. PET/CT and biopsies are second highest volume.

Many patients are also placed in a surveillance program and return for multiple imaging exams over the course of their IF. For example, a lung nodule < 8 mm is imaged annually or more frequently to monitor growth or until stable for two years per Fleischer criteria. The increase in imaging volume as a result of the FIND Program more than covers the cost of the nurse navigator staff in the FIND Program. Increased volume in oncology with cancer diagnosed, pulmonary medicine, and procedures are also attributed to the FIND Program. The number of lung IF and cancers identified is the highest volume in the program.

The software was purchased in 2017 with institutional grant funding, with a focus on incidental lung nodules only—with annual volume at 3000 exams. In 2019, we received another institutional grant to expand the FIND Program to include all incidental findings (all body systems). Annually volume increased to 6000 exams. In 2020, the software vendor added artificial intelligence natural language understanding software to enhance the IF capture and the annual volume increased to 19,000 exams annually. This is for Southeast Michigan with an annual total radiology report volume of 750,000. Southeast Michigan continues at 19,000 annual IFs captured.

The software is robust and can be utilized for searches in other settings, including for other reports such as in cardiology and laboratory services. For example, we are exploring small opportunities in interventional radiology that would aid in handling manual processes and ensure better coverage.

The program could be housed elsewhere, but the in-depth knowledge of the reports, continuous improvement, enhanced opportunities, and associated revenue to more than cover the cost are strong support.

The FIND Business Plan developed by the institutional finance department demonstrates the program covers the program costs and reports a profit. More importantly, the measurable benefits of the FIND Program include the lives extended, patients retained in the healthcare system, and reduced medicolegal risk and settlements for physicians and the health system.
