# Correction to: The FIND Program: Improving Follow-up of Incidental Imaging Findings

**DOI:** 10.1007/s10278-023-00898-7

**Published:** 2023-09-15

**Authors:** Kaitlin M. Zaki-Metias, Jeffrey J. MacLean, Alexander M. Satei, Serguei Medvedev, Huijuan Wang, Christopher C. Zarour, Paul J. Arpasi

**Affiliations:** 1 Department of Radiology, Trinity Health Oakland Hospital/Wayne State University School of Medicine, Pontiac, MI USA; 2 Huron Valley Radiology, Ypsilanti, MI USA

**Correction to: The FIND Program: Improving Follow-Up of Incidental Imaging Findings** 10.1007/s10278-023-00780-6

There was a typographical error in the number of studies analyzed prior to and after implementation of the FIND Program quoted in the Materials & Methods and Results sections.

The first sentence of the second paragraph of the Materials & Methods section should read: “Electronic medical records and final reports of a total of 2000 randomly selected patients were investigated. A total of 1000 patients with 1274 studies from January 2019 to January 2020 prior to implementation of the FIND Program and 1000 patients with 1228 studies from September 2020 to September 2021, 1 year after implementation of the FIND Program were analyzed.”

The first sentence of the first paragraph of the Results section should read: “A total of 1274 studies were performed on 1000 patients from January 2019 to January 2020 prior to implementation of the FIND Program at Trinity Health Oakland Hospital, and 1228 studies were performed on 1000 patients from September 2020 to September 2021, 1 year after implementation of the FIND Program.”

There was a typographical error in the number and percentage of completed recommended follow-up imaging in the pre-implementation group in Table 1 in the original submission. Please see the corrected Table 1 below.

**Table 1:** Frequency of incidental finding detection, follow-up recommendation, and completed follow-up prior to and after implementation of the FIND Program

	***n*/*N***	**%**	***p*-value**
**Frequency of detection of incidental imaging findings**			
Pre-implementation	82/1274	6.4%	0.452
Post-implementation	70/1228	5.7%	
**Frequency of follow-up recommendations**			
Pre-implementation	52/69	75.4%	0.001
Post-implementation	67/70	95.7%	
**Completion of recommended follow-up imaging**			
Pre-implementation	16/52	30.7%	0.03
Post-implementation	34/67	50.7%	

The original article has been corrected.

